# Inter-year consistencies and discrepancies on intestinal microbiota for overwintering relict gulls: correlations with food composition and implications for environmental adaptation

**DOI:** 10.3389/fmicb.2024.1490413

**Published:** 2024-12-09

**Authors:** Hong Wu, Hongyu Yao, Menglin Sun, Ran Wang, Zeming Zhang, Nan Wu, Dapeng Zhao

**Affiliations:** College of Life Sciences, Tianjin Normal University, Tianjin, China

**Keywords:** migratory birds, high-throughput sequencing technology, fecal microscopy, diet composition, intestinal microbiota

## Abstract

The gut microbiota of migratory birds is influenced by their food choices, and exploring the potential relationship between diet composition and gut microbiota can help better protect related species. By integrating non-invasive sampling techniques, high-throughput sequencing technology, and microscopic examination technology, this study presents the first evidence on diet composition during overwintering periods as well as the potential relationship between diet composition and gut microbiota in wild relict gulls (*Larus relictus*). Thirty-five fecal samples from two consecutive overwintering periods (2021 and 2022 overwintering periods) in Tianjin coastal wetland were used to investigate inter-year consistencies and discrepancies on diet composition and gut microbiota in wild *Larus relictus.* It was found that the common dominant phyla of both 2021 and 2022 group included Firmicutes, Proteobacteria, Chloroflexi and Actinobacteriota. The common dominant genera were *Catellicoccus* and *Ilumatobacter*. The diversity of gut microbiome in 2022 group was higher, while the richness was not significantly different. Based on the high-throughput sequencing technology of 18S rDNA, the study found that the dominant classes within the diet components of *Larus relictus* included Polychaeta, Bivalvia, Malacostraca, Gastropoda, unclassified_p__Dinoflagellata, Dinophyceae, and Ostracoda. Among them, Bivalvia, Malacostraca, and Gastropoda were also found with microscopic examination technology from the same samples. The abundance of Fusobacteriota and *Cetobacterium* were positively correlated with the abundance of Bivalvia and Malacostraca; while the abundance of *Psychrobacter* and *Breznakia* were negatively correlated with the abundance of Malacostraca and Gastropoda. Findings from this study could provide scientific references for health monitoring and conservation of relict gulls.

## Introduction

The gut microbiome affects the metabolism and health of animals, meanwhile the dietary structure directly affects the composition of gut microbiome (Adak and Khan, 2019). Currently, high-throughput sequencing technology based on fecal samples help us deepen our understanding of the characteristics of wildlife gut microbiome (Hermann-Bank et al., 2013). Fecal samples are the most popular and convenient sample type, which have significant advantages since it is noninvasive for focal endangered species (Kophamel et al., 2022). The gut microbiome of many wild animals across taxa had been studied through 16S rRNA gene sequencing, such as Sichuan hill partridge (*Arborophila rufipectus*) (Ma et al., 2023), Oriental stork (*Ciconia boyciana*) (Wu et al., 2021), and Malayan tapir (*Tapirus indicus*) (Arumugam et al., 2023). The gut microbiome composition of wildlife, particularly avian species, is influenced by external factors (e.g., climate, habitat environment, behavioral characteristics) and internal factors (e.g., diet, digestive tract morphology, sex) (Waite and Taylor, 2015). Migratory birds experience significant changes in their habitat conditions during different life stages (Cao et al., 2020). During migration process in spring and autumn, wild birds often acquire food from diverse stopover sites, while in summer and winter, they primarily rely on food sources at relatively stable breeding site and overwintering site, respectively, to meet energy and nutritional requirements (Salewski and Bruderer, 2007). Tang et al. compared the gut microbiota in the Sichuan partridges across three critical periods of their annual life cycle (Tang et al., 2023). Dong et al. used high-throughput sequencing technology to sequence the gut microbiota of *A. cerana* at different developmental stages (Dong et al., 2021). Therefore, continuous monitoring on gut microbiota for migratory birds, especially for endangered species, can enhance our understanding of variation on its gut microbiome and further facilitate its health assessment and scientific conservation.

Food choice is an important research field in conservation biology, and research methods on wildlife diet composition are constantly being enriched due to variation in related technology and feasible applications (Deagle et al., 2007). Each method for wildlife dietary composition has certain advantages and limitations (Gosselin et al., 2017). For instance, direct observation method can accurately determine food category consumed by the target species, but this method is time-consuming and difficult to carry out continuously day and night, especially for birds (Russo et al., 2024). Therefore, given the advantages and disadvantages of each research method, it is necessary to apply two or more research methods simultaneously in the study related to dietary composition of wild animals (Oehm et al., 2011; Schumm et al., 2023). Nowadays methods applied for wildlife dietary composition mainly include direct observation (Hall et al., 2000), gastric content analysis (Lance and Thompson, 2005), microscopic examination technology (Samelius and Alisauskas, 1999), food residue collection (Hayakawa et al., 2018), stable isotope analysis (Ogden et al., 2005), and high-throughput sequencing technology (Correia et al., 2023). Among these methods, both microscopic examination technology and high-throughput sequencing technologies, with the non-invasive characteristic, are often chosen as the popular methods for diet analysis across various animal taxa including bar-tailed godwit (*Limosa lapponica*) (Scheiffarth, 2001), black-headed gull (*Larus ridibundus*) (Iwamatsu et al., 2007); Steller sea lion (*Eumetopias jubatus*) (Deagle et al., 2007). However, up to now, detailed information on dietary composition of wildlife, especially endangered migratory birds, is relatively limited. Accurate and comprehensive knowledge of diet analyses can be helpful to understand its ecological requirement and provide effective conservation strategies for endangered species (Zhao et al., 2022; Hanson et al., 2021).

Relict gulls (*Larus relictus*) belong to the Laridae family within the Charadriiformes order. It is classified as “vulnerable” species by the International Union for Conservation of Nature (IUCN) and listed as one first-class protected bird in China (BirdLife International, 2024; Wang et al., 2022). The breeding grounds of relict gulls are relatively scattered, mainly located in Hebei Province, Inner Mongolia Autonomous Region, and Shaanxi Province of China (Cardoso et al., 2016). The Tianjin coastal wetland located around the Bohai Bay is an important overwintering site for wild relict gulls (Liu et al., 2006).To date the current research on the diet of wild *L. relictus* mainly comes from the breeding site (Wang et al., 2013; Liu et al., 2017), whereas the composition of their diet during the overwintering place has not been reported. On the contrary, apart from geographical variations, the gut microbiota of wildlife undergoes temporal changes (such as seasonal fluctuations) and adaptations to physiological requirements. Yao et al. had compared the intestinal flora at different overwintering periods (Early and late stage) in wild relict gulls (Yao et al., 2023). Huang et al. used the metagenomic sequencing to characterize and compare the community composition and antibiotic resistance of the gut microbiota from relict gulls and *Anatidae* species from 2021 to 2023 (Huang et al., 2024).

This study applies both high-throughput sequencing technology and microscopic examination technology to present the first evidence of diet composition of wild relict gulls during overwintering periods, and for the first time explore the potential relationship between diet composition and gut microbiota in this species. Data from two consecutive overwintering periods were used to investigate inter-year consistencies and discrepancies on diet composition and gut microbiota in *L. relictus*, which findings could help for the formulation of comprehensive conservation and management policies for this species and its overwintering area.

## Materials and methods

### Sample collection

In this study, 21 and 14 fresh fecal samples of wild relict gulls were collected from Tianjin Relict Gull Park in China during 2021–2022 and 2022–2023 overwintering periods, respectively. In the wild, we maintained the safe distance from focal species, and used binoculars to observe individual behavior. After they defecated and leaved their original habitats, we promptly rushed to the place where they just stopped to collect fecal samples as soon as possible. Fecal sample collected from 2021 to 2022 overwintering period and 2022–2023 overwintering period were named as 2021 group and 2022 group, respectively (Supplementary Table S1). All fecal samples were placed in sterile 5 mL EP tubes and transported back to the laboratory as soon as possible, then stored at −80°C.

### DNA extraction, library preparation, and 16S rRNA sequencing

The total DNA was extracted from fecal samples using the QIAamp DNA Stool Kit (QIAGEN, Germany) following the manufacturer’s instructions. After the total DNA in the fecal samples was extracted, the absorbance ratios of A260/A230 and A260/A280 were measured using a NanoDrop 2000 spectrophotometer (ThermoFisher Scientific, Wilmington, DE, USA) to evaluate the quality. The final extracted product was measured for DNA concentration using a Qubit 2.0 fluorometer (Life Technologies, Carlsbad, CA, USA). Sequencing of the 16S rRNA (V3-V4 region) was performed using universal primers 338F 5′-ACTCCTACGGGA GGCAGCA-3′ and 806R 5′-GGACTACHVGGGTWTCTAAT-3′ (Fadrosh et al., 2014). The PCR products were detected by 1% agarose gel electrophoresis and purified by the AxyPrep DNA Gel Recovery Kit (AXYGEN Corporation, Silicon Valley, CA, USA). Then, the PCR amplification products were conducted using the Illumina MiSeq platform at Shanghai Meiji Bio-Pharmaceutical Technology Co., Ltd. (Shanghai, China).

### Bioinformatics and statistical analyses of gut microbiome

Bioinformatics analysis of the sequence data was performed using the QIIME2 (version 2021.02) software package (Hall and Beiko, 2018; Bolyen et al., 2019). Raw sequence data were filtered, dereplicated, and denoised using DADA2 as implemented in QIIME2 (Callahan et al., 2016). The taxonomy profile of OTU was generated using the SILVA database (Quast et al., 2013). In this study, the phylum and genus with abundance more than 1% among fecal samples were defined as the dominant phylum and genus respectively, and inter-group differences on dominant phyla and genera were analyzed using Wilcoxon rank sum tests.

The alpha diversity of the microbiome was measured using Ace index, Chao index, Shannon index, and Simpson index based on the number of observed OTUs. The Ace and Chao indices reflect community abundance, while the Shannon and Simpson indices reflect community diversity. The significance of inter-group differences in alpha diversity was evaluated using Wilcoxon rank sum tests. The beta diversity of gut microbiota was calculated based on weighted and unweighted UniFrac distances (Lozupone and Knight, 2005), and principal coordinates analysis (PCoA) plots were used to visualize the inter-group difference (Vazquez-Baeza et al., 2013).

### Diet analysis based on high-throughput sequencing technology

The V4 region of the 18S rRNA gene was amplified to detect the eukaryotic communities in the gut contents of relict gulls from both groups using universal primers TAReuk454FWD1 5’-CCAGCASCYG CGGTAATTCC-3′ and TAReukREV3 5’-ACTTTCGTTCTTGA TYRA-3′. Thermal cycling conditions of amplification were: 3 min of denaturation at 94°C, followed by 27 cycles of 30 s at 94°C, 30 s at 55°C, 30 s at 72°C and a final extension at 72°C for 5 min. PCR reactions were performed in triplicate 50 μL mixture and PCR products were extracted and purified using the AxyPrep DNA Gel Extraction Kit (Axygen Biosciences, Union City, CA, USA) according to the manufacturer’s protocol. Purified amplicons were pooled in equimolar and paired-end sequenced on an Illumina Miseq platform (Illumina, San Diego, USA) at Shanghai Meiji Bio-Pharmaceutical Technology Co., Ltd. (Shanghai, China).

Raw FASTQ files were demultiplexed, quality-filtered and merged. It was necessary to remove the sequences of relict gulls themselves before analysis. After sequence screening, operational taxonomic units (OTUs) were clustered with a 97% similarity cutoff using UPARSE (version 7.11) and chimeric sequences were identified and removed using UCHIME (Edgar et al., 2011). The taxonomy of each OTU representative sequence was analyzed using RDP Classifier algorithm2 against the NCBI nucleotide sequence database NT (NT_v20200327/18S_eukaryota). Finally, the corresponding species information of each OTU was obtained. Rarefaction curves were plotted by using Mothur for each sample.

### Diet analysis based on microscopic examination technology

In this study, 0.3 g for each fecal sample was soaked in saturated biological enzyme detergent for more than 24 h. Three microscopic slides were prepared for each sample, which were observed with a Nikon SMZ25 microscope (Nikon, Tokyo, Japan). During the observation, the shape, color, etc. of animal remains in the field of vision and the frequency they appeared were all recorded. Each sample was randomly photographed 50 times. The species from fecal samples were mainly identified based on their typical characteristics (such as the shell patterns of bivalve species and the pincers of crustacean species).

The frequency of each type of food in the sample was expressed as *F* = the number of times a certain type of food appears in the sample/the total number of samples ×100%. The frequency *F* was converted into the average density of each type of food:


$$D=-\ln\left(1-F/100\right)$$

The average density *D* can be further converted into relative density (*RD*), used as the metric for quantifying the food composition, which was calculated according to the following equation:


$$RD_{i}=D_{i}/\Sigma D_{i}\times 100$$

### Relationship between diet composition and gut microbiota

SPSS 26.0 software was applied for statistical analysis to explore the potential correlation between diet composition and dominant phyla/genera identified from gut microbiota based on Spearman’s rank coefficient. Correlation plots were constructed by means of Origin 2023b software. The significance levels in statistical analysis within the present study were set at three levels (0.05, 0.01, and 0.001). The symbol * in the figure indicates 0.01 ≤ *p* < 0.05, which was considered as statistically significant. The symbol ** in the figure indicates 0.001 ≤ *p* < 0.01, which was considered as extremely statistically significant. The symbol *** in the figure indicates *p* ≤ 0.001, which was considered as super statistically significant.

## Results

### The composition of gut microbiome

After Illumina MiSeq sequencing, a total of 1,757,185 optimized sequences were obtained, with an average length of 420 bp. After clustering at 97% similarity, a total of 2,551 OTUs were obtained, including 44 phyla, 111 classes, 247 orders, 398 genera, 758 species, and 1,261 strains. Among them, 43 phyla, 106 classes, 225 orders, 349 genera, 639 species, and 1,033 strains were obtained from the 2021 group. Meanwhile, 38 phyla, 92 classes, 209 orders, 333 genera, 611 species, and 983 strains were obtained from the 2022 group. There were 1,306 OTUs shared by both groups. Seven hundred and four and 541 OTUs were unique in 2021 group and 2022 group, respectively.

**Figure 1:**
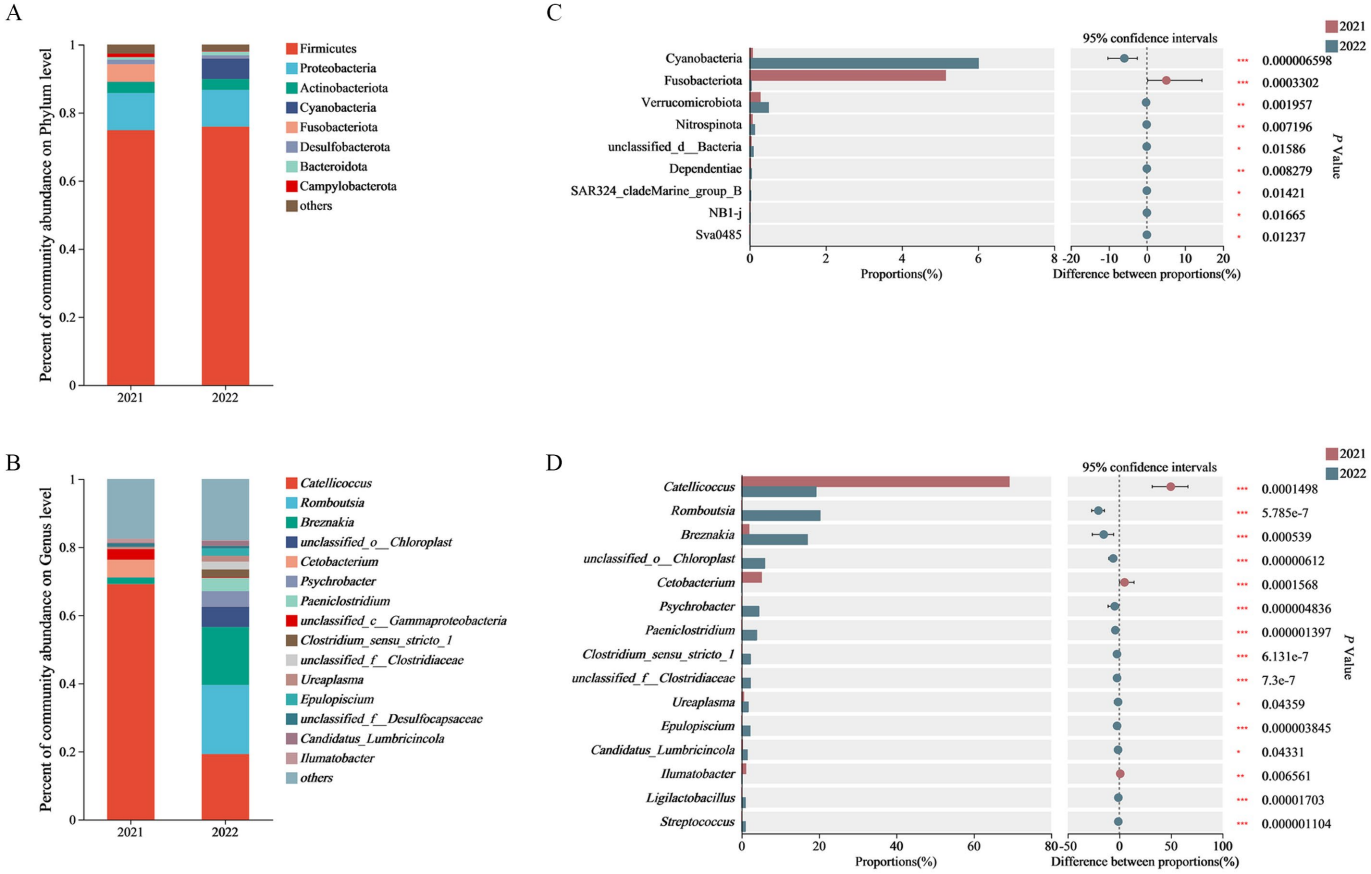
Phylum-level **(A)** and genus-level **(B)** composition of gut microbiome as well as phylum-level **(C)** and genus-level **(D)** inter-group comparison on abundance of gut microbiome.

The dominant bacterial phyla in 2021 group were Firmicutes (74.87%), Proteobacteria (10.90%), Fusobacteriota (5.14%), Actinobacteriota (3.26%), Desulfobacterota (1.34%) and Bacteroidota (1.06%). The dominant bacterial phyla in 2022 group were Firmicutes (75.92%), Proteobacteria (10.78%), Chloroflexi (6.01%), Actinobacteriota (3.21%) and Bacteroidota (1.11%). The common dominant bacterial phyla of both groups were Firmicutes, Proteobacteria, Chloroflexi and Actinobacteriota. The unique dominant bacterial phyla in 2021 group were Fusobacteriota and Desulfobacterota, while the unique dominant bacterial phyla in 2022 group was Bacteroidota (Figure 1A and Supplementary Table S2).

The dominant bacterial genera in 2021 group included *Catellicoccus* (69.15%), *Cetobacterium* (5.13%), *unclassified_c__Gammaproteo bacteria* (3.18%), *Breznakia* (1.90%), *Ilumatobacter* (1.09%), and *unclassified_f_Desulfocapsaceae* (1.03%). The dominant bacterial genera in 2022 group included *Romboutsia* (20.29%), *Catellicoccus* (19.26%), *Breznakia* (17.01%), *unclassified_o__Chloroplast* (5.99%), *Psychrobacter* (4.49%), *Paeniclostridium* (3.90%), *Clostridium_Sensu_stricto-1* (2.30%), *unclassified_f_Clostridiaceae* (2.27%), *Ureaplasma* (1.72%), *Epulopiscium* (2.19%), and *Candidatus_Lumbricincola* (1.48%). The common dominant bacterial genera of both groups were *Catellicoccus* and *Ilumatobacter*. The unique dominant bacterial genera in 2021 group included *Cetobacterium*, *unclassified_c__Gammaproteobacteria*, *Breznakia*, and *unclassified_f_Desulfocapsaceae*, whereas the unique dominant bacterial genera in 2022 group included *Romboutsia*, *unclassified_o__Chloroplast*, *Psychrobacter*, *Paeniclostridium*, *Clostridium_Sensu_stricto-1*, *unclassified_f_Clostridiaceae*, *Ureaplasma*, *Epulopiscium*, *and Candidatus_Lumbricincola* (Figure 1B and Supplementary Table S3).

Based on Wilcoxon rank sum tests, the abundance of the Fusobacteriota in 2021 group was significantly higher than that in 2022 group on the phylum level. The abundance of *Catellicoccus*, *Cetobacterium* and *Ilumatobacter* in 2021 group was significantly higher than that in 2022 group on the genus level. Meanwhile, the abundance of *Romboutsia*, *Breznakia*, *unclassified_o_Chloroplast*, *Psychrobacter*, *Paeniclostridium*, *Clostridium_sensu_stricto_1*, *unclassified_f_Clostridiaceae*, *Epulopiscium*, *Ligilactobacillus*, and *Streptococcus* in 2022 group was super significantly higher than that in 2021 group (Figures 1C,D).

### Inter-group comparison on alpha and beta diversity

**Figure 2:**
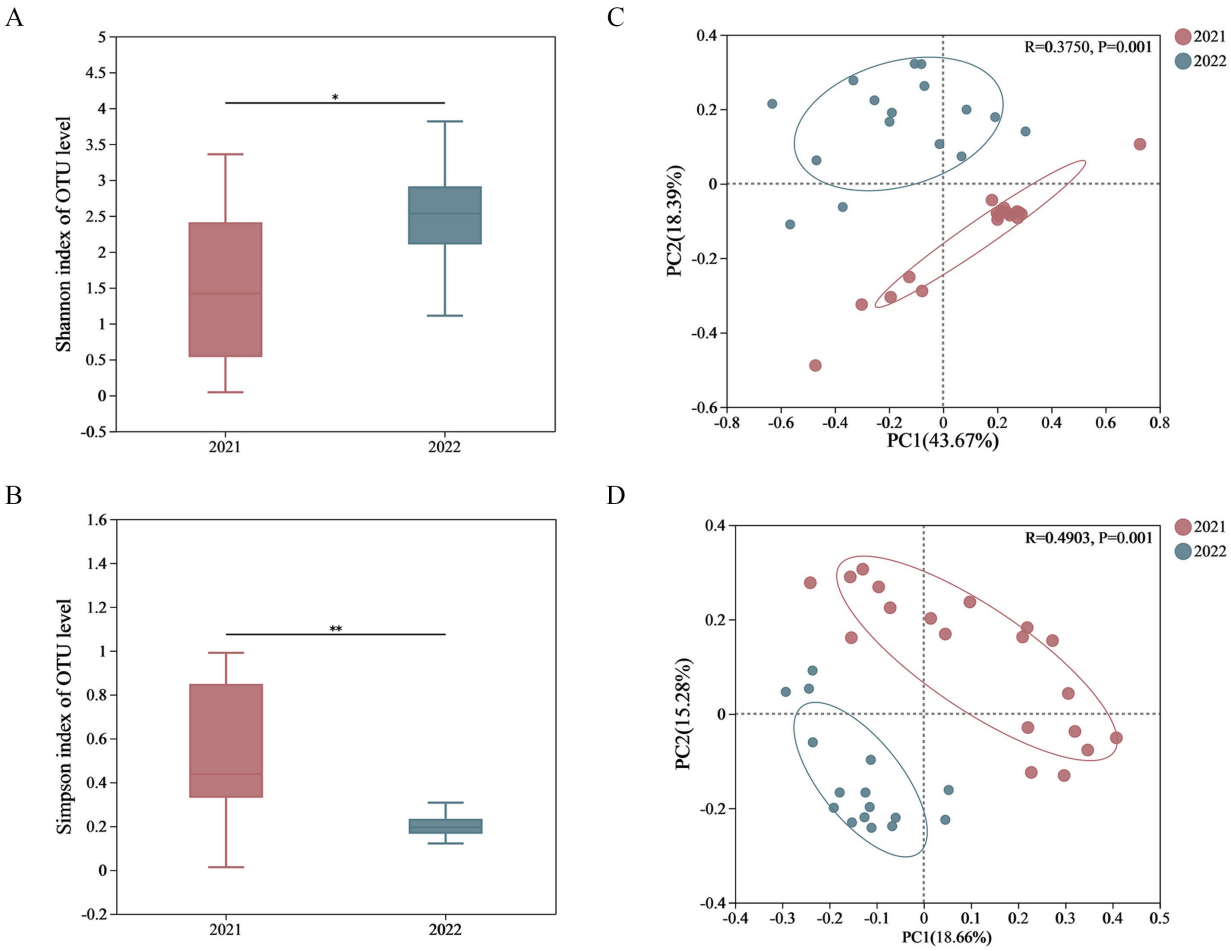
Inter-group comparison on the alpha and beta diversity. The alpha diversity was calculated and showed significant differences in Shannon **(A)** and Simpson **(B)** indexes; the PCoA was used to evaluate the beta diversity on weighted Unifrac distances **(C)** and unweighted Unifrac distances **(D)**.

The curve trends for all samples were similar, thus two groups had similar abundance and uniformity in terms of gut microbiota (Supplementary Figure S1). The alpha diversity index of fecal microbial composition including Chao’s index, Ace’s index, Simpson’s index, and Shannon’s index were calculated (Supplementary Table S4). The Shannon index of 2022 group was significantly higher than that of 2021 group, while the Simpson indexes of 2022 group was significantly lower. There was no significant inter-group difference on both Ace index and Chao index, indicating that the diversity of gut microbiome in 2022 group was higher than that in 2021 group, while the richness was not significantly different (Figures 2A,B and Supplementary Figure S2).

The PCoA was used to evaluate the beta diversity of fecal microbial composition (Figures 2C,D). For 2021 group and 2022 group, the contribution rates of PC1 and PC2 were 43.67 and 18.39%, respectively, based on weighted Unifrac distances, whereas the contribution rates of PC1 and PC2 were 18.66 and 15.28%, respectively, based on unweighted Unifrac distances. There was a significant inter-group difference for both weighted_unifrac (*p* = 0.001) and unweighted_unifrac (*p* = 0.001), leading the complete separation.

### Diet composition based on high-throughput sequencing technology

DNA was extracted from relict gulls’ feces, and a database of fecal contents was established based on the results of 18S rRNA V4 amplification regions. The food composition of both groups was very similar, but there were certain inter-group differences on the relative proportions of some diets. Rarefaction curves for all samples were nearly saturated, suggesting sufficient sequencing depth for this study (Supplementary Figure S4). High-throughput sequencing of 18S rRNA V4 region yielded 715,818 clean sequences from all fecal samples.

**Figure 3:**
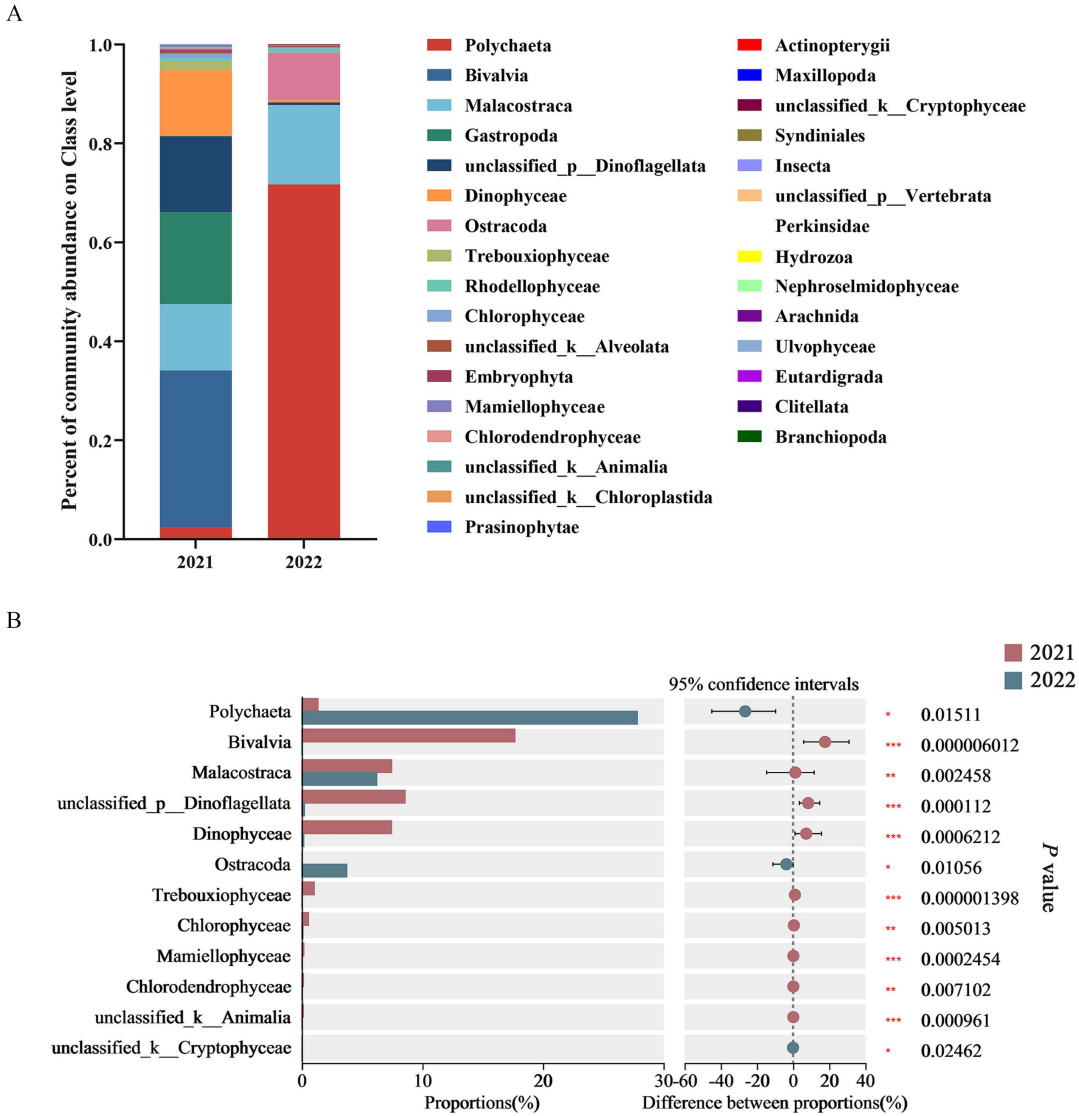
Class-level diet composition from fecal samples **(A)** and inter-group comparison on their abundance **(B)** based on current findings from high-throughput sequencing technology of the 18S rRNA.

After blasting against NCBI using BLASTN and removing sequences of relict gulls, fungi, and parasites, the DNA sequences of gut contents were mainly classified at the class level into Polychaeta, Bivalvia, Malacostraca, Gastropoda, unclassified_p__Dinoflagellata, Dinophyceae, and Ostracoda. Based on high-throughput sequencing technology, this study found that dominant classes in 2021 group included Bivalvia (31.71%), Polychaeta (2.41%), Malacostraca (13.37%), Gastropoda (18.62%), unclassified_p__Dinoflagellata (15.36%), Dinophyceae (13.35%), and Trebouxiophyceae (1.85%); while dominant classes in 2022 group included Polychaeta (71.72%), Malacostraca (16.02%), and Ostracoda (9.60%) (Figure 3A).

Based on Wilcoxon rank sum tests, the abundance of Bivalvia, unclassified_p_Dinoflagellata, Dinophyceae, Trebouxiophyceae, Mamiellophyceae, unclassified_k_Animalia, Malacostraca, Chlorophyceae and Chlorodendrophyceae in 2021 group was significantly higher than that in 2022 group. Meanwhile, the abundance of Polychaeta, Ostracoda, and unclassified_k_Cryptophyceae in 2022 group was significantly higher than that in 2021 group (Figure 3B).

### Diet composition based on microscopic examination technology

**Figure 4:**
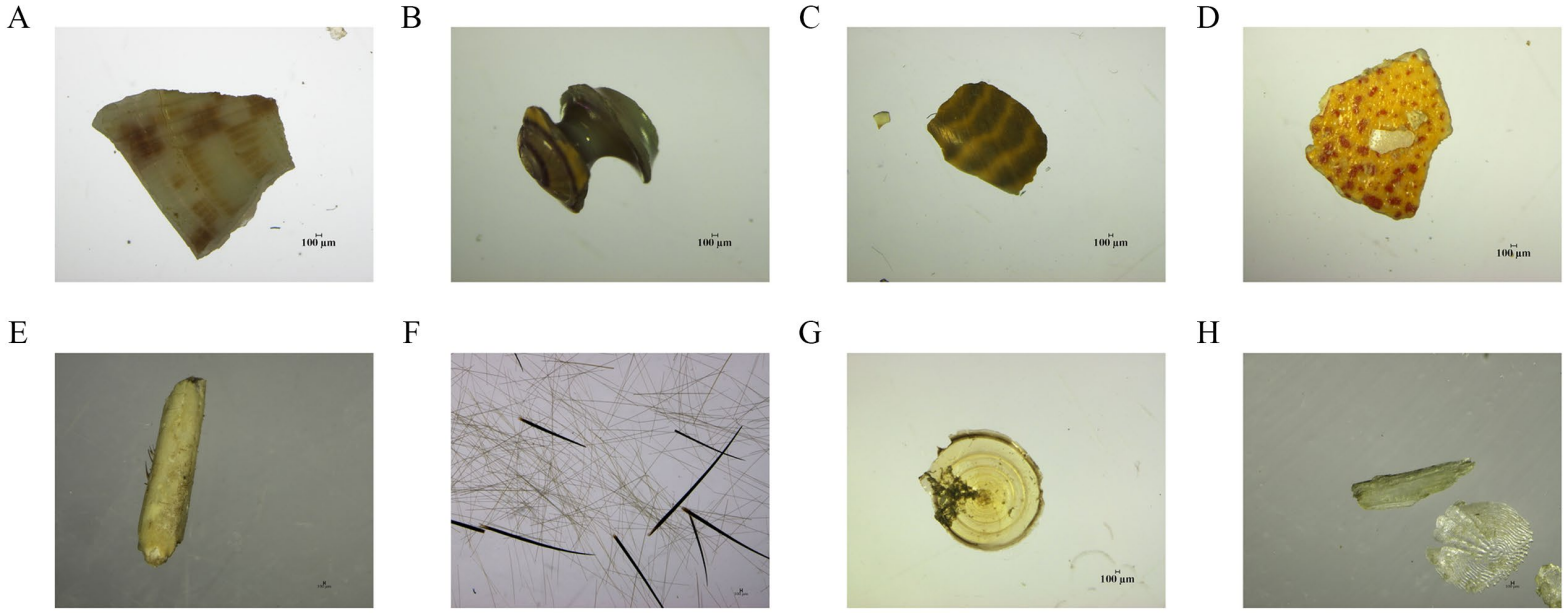
Representative residual of the diet composition under microscopic examination technology on the class level. **(A)** Bivalvia; **(B,C)** Gastropoda; **(D,E)** Malacostraca; **(F)** Polychaeta; **(G,H)** Osteichthyes.

Based on microscopic examination technology, this study found that: (1) the diet for 2021 group mainly consisted of species from Bivalvia, Gastropoda, Malacostraca, and Osteichthyes on the class level, and its identified species included *Ruditapes philippinarum*, *Scapharca kagoshimensis*, *Mactra veneriformis*, *Potamocorbula laevis*, *Solen strictus*, *Umbonium tomasi* and *Nassarius festivus*; (2) the diet for 2022 group mainly consisted of species from Bivalvia, Gastropoda, Malacostraca, Osteichthyes and Polychaeta on the class level, and its identified species also included *Ruditapes philippinarum*, *Scapharca kagoshimensis* similar with the result from 2021 group; (3) the commonly found classes of both groups included Bivalvia and Gastropoda, in which the former class owned the highest proportion. The proportion of Bivalvia in 2022 group (56.06%) has decreased compared to that in 2011 group (58.68%). Compared with 2021 group, Polychaeta appeared for the first time in 2022 group and accounted for a large proportion (18.36%) (Table 1 and Figure 4).

**Table 1:** Class-level diet composition based on microscopic examination technology in each overwintering year.

Group name	Class name	*F*(%) mean	*D_i_*mean	*RD_i_*(%) mean
2021 group	Bivalvia	28.53	0.3629	58.68
	Gastropoda	4.11	0.0485	11.69
	Malacostraca	7.37	0.0948	23.86
	Osteichthyes	0.11	0.0011	0.19
	others	1.79	0.0189	3.40
2022 group	Bivalvia	16.13	0.1830	56.06
	Gastropoda	0.63	0.0066	1.29
	Malacostraca	1.38	0.0141	4.16
	Osteichthyes	0.13	0.0013	0.13
	Polychaeta	12.38	0.2114	18.36
	others	3.75	0.0455	20.00

### Relationship between diet composition and gut microbiota

**Figure 5:**
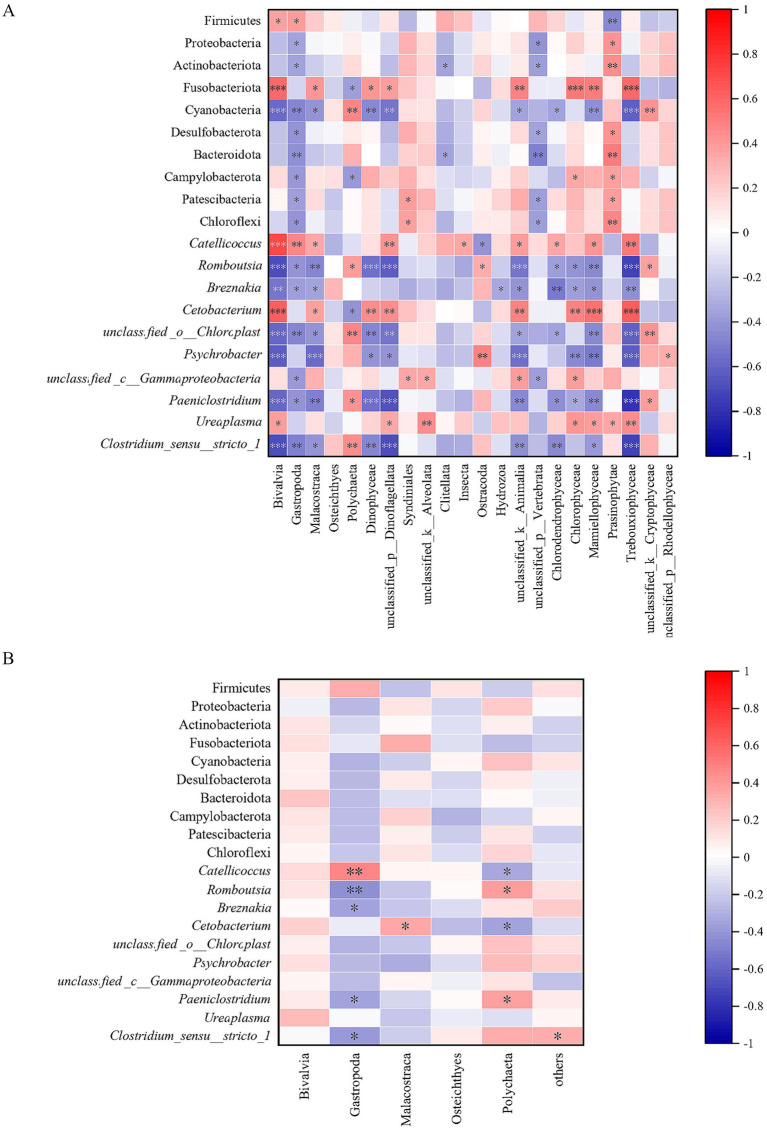
The relationship between diet composition and gut microbiota based on high-throughput sequencing technology **(A)** and microscopic examination technology **(B)**.

Spearman’s rank correlation analysis showed that the main foods of relict gulls had an extremely significant correlation with intestinal bacteria (Figure 5). With regard to dietary findings from high-throughput sequencing technology, it was found that: (1) the abundance of Bivalvia was significantly positively correlated with that of Firmicutes and *Ureaplasma*; the abundance of Bivalvia was super significantly positively correlated with that of Fusobacteriota, *Catellicoccus*, and *Cetobacterium*; (2) the abundance of Gastropoda was significantly positively correlated with that of Firmicutes; the abundance of Gastropoda was extremely significantly positively correlated with that of *Catellicoccus*; (3) the abundance of Malacostraca was significantly positively correlated with that of Fusobacteriota, *Catellicoccus*, and *Cetobacterium*; (4) the abundance of Polychaeta was significantly positively correlated with that of *Romboutsia* and *Paeniclostridium*; the abundance of Polychaeta was extremely significantly positively correlated with that of Cyanobacteria, *unclassified_o_Chloroplast* and *Clostridium_sensu_stricto_1*; (5) the abundance of Dinophyceae was significantly positively correlated with that of Fusobacteriota; the abundance of Dinophyceae was extremely significantly positively correlated with that of *Cetobacterium* (Figure 5A).

With regard to dietary findings from microscopic examination technology, it was found that: (1) the abundance of Gastropoda was extremely significantly positively correlated with that of *Catellicoccus*; (2) the abundance of Gastropoda was extremely significantly negatively correlated with that of *Romboutsia*, and significantly negatively correlated with that of *Brznakia*, *Paeniclostridium* as well as *Clostridium_sensu_stricto_1*; (3) the abundance of Malacostraca was significantly positively correlated with that of *Cetobacterium*; (4) the abundance of Polychaeta was significantly negatively correlated with that of both *Romboutsia* and *Paeniclostridium* (Figure 5B).

## Discussion

### Spatiotemporal differences on intestinal microorganisms

Based on high-throughput sequencing, this study analyzed intestinal microbiota of relict gulls from the same overwintering area in different years. The results showed that in both 2021 group and 2022 group, the Firmicutes and Proteobacteria were two main common dominant phyla of the overwintering relict gulls. This is consistent with previous related studies of other species from Laridae family (e.g., black-headed gull; Laviad-Shitrit et al., 2019; Liao et al., 2019) as well as Charadriiformes order (e.g., red knot; Cho and Lee, 2020). Firmicutes can help host organisms break down complex carbohydrates, polysaccharides, and fats, thereby improving the ability of host organisms to absorb energy and nutrients from daily food (Sun et al., 2023). Proteobacteria have multiple physiological functions and can help meet the higher energy and nutrient requirements of organisms by utilizing carbon sources (Sharma et al., 2022).

There is limited research on intestinal microbiota of relict gulls. Zhao et al. (2023) compared the gut bacteria of relict gulls and black-necked grebe in Erdos Relic Gull National Nature Reserve in Inner Mongolia, China (Zhao et al., 2023), and found that the most abundant bacterial phyla included Proteobacteria, Firmicutes, Clostridium, and Bacteroidota. Liu et al. (2022) focused on the characteristics of the intestinal microbiota of *L. relictus* during the breeding period in Inner Mongolia. Firmicutes and Proteobacteria were the most abundant microbiota in relict gulls (Liu et al., 2022). Our group has previously reported on the characteristics of intestinal microbiota of relict gulls at different stages during overwintering periods (Yao et al., 2023). Comparing intestinal microbiota of *L. relictus* in the overwintering period and breeding period (Liu et al., 2022), the common dominant phyla included Firmicutes, Bacteroidota, Actinobacteriota, and Proteobacteria. The unique dominant phyla in this study during overwintering were Actinobacteriota and Desulfobacterota, while the unique dominant phyla in breeding period from previous study (Liu et al., 2022) were Verrucomicrobiota and Planctomycetes. The common dominant genera included *Catellicoccus*, *Cetobacterium*, *Paeniclostridium*, and *Clostridium_sensu_stricto_1*. The unique dominant genera during overwintering period in this study included *Romboutsia*, *Breznakia*, *unclassified_o__Chloroplast*, *Psychrobacter*, *unclassified_c__Gammaproteobacteria*, *Ureaplasma*, *unclassified_f_Clostridiaceae*, and *Epulopiscium*. The unique dominant genera during breeding period included *Escherichia-Shigella*, *Lactobacillus*, *uncultured_bacterium_f_Enterobacteriaceae*, *Enterococcus*, and *Mycoplasma* (Liu et al., 2022). The presence of some unique phyla and genera at two different studies on the intestinal microbiota of *L. relictus* may be related to differences in their diet compositions during overwintering and breeding periods. For example, Desulfobacterota is a unique dominant phylum in this study, while *Psychrobacter* and *Breznakia* are unique dominant genera in this study.

In addition to comparing the differences in gut microbiota between the overwintering period and the breeding period, we also further explored the inter-year difference on gut microbiota of this species from a spatial and temporal perspective. According to previous reports, different spatial and temporal dimensions can cause differences and changes of gut microbiota, which may also be a strategy for animals to adapt to environmental changes. For example, Eddington et al. (2021) reported the spatial and temporal variation of the microbial community in the feces of mule deer (*Odocoileus hemionus*), and revealed that this change may be associated with indicators of host health (Eddington et al., 2021). Perofsky et al. (2021) conducted a continuous survey of wild Verreaux’s sifaka (*Propithecus verreauxi*) for 5 years, and found that six social groups maintained distinct gut microbial characters. The gut samples from group members during each season exhibited greater similarity compared to these from single individuals across different years (Perofsky et al., 2021).

When comparing intestinal microbiota of *L. relictus* in different overwintering years, the abundance of Actinobacteriota and *Cetobacterium* in 2021 group was significantly higher than that in 2022 group. Previous studies have shown that intestinal microbiota of carnivorous birds usually has a higher abundance of Actinobacteriota (Florez et al., 2015; Lewin et al., 2016). Cetobacterium, which belongs to the Actinobacteriota, is involved in lipid metabolism and identified as a gut microbe in various freshwater fish (Larsen et al., 2014). It has been reported that the abundance of *Cetobacterium* was significantly higher in the fish-eating birds than in the other birds (Xiao et al., 2021). Since food eaten by relict gulls contain fish, food-derived microbes may affect the gut microbiome, thus we speculate that the enriched level of *Cetobacterium* in these birds might be directly from fish intake, and intake of fish in 2022 group was higher than that in 2021 group. Our study found that the abundance of Bivalvia and Malacostraca in 2021 group was higher than that in 2022 group. Based on Spearman’s rank correlation analysis, the abundance of both Actinobacteriota and *Cetobacterium* is positively correlated with the abundance of both Bivalvia and Malacostraca. Therefore, we speculate that the inter-group difference on the abundance of both Actinobacteriota and *Cetobacterium* within gut microbiota of *L. relictus* may be related to the composition of food intake and related nutritional components during the overwintering period. Cyanobacteria is a type of prokaryotic organism that undergoes photosynthesis symbiotically with fungi (Zhou et al., 2024), and this study found that the abundance of Cyanobacteria in 2022 group was significantly higher than that in 2021 group.

### Methodological comparison on diet composition

In this study, both high-throughput sequencing technology and microscopic examination technology from gut contents were used to investigate diet composition of relict gulls, and different methods yielded consistency and differences. For example, based on high-throughput sequencing of 18S rRNA, the dominant orders in the diet of relict gulls included Polychaeta, Bivalvia, Malacostraca, Gastropoda, unclassified_p__Dinoflagellata, Dinophyceae, and Ostracoda, while fecal microscopy found that the dominant orders in the diet included Bivalvia, Malacostraca, and Gastropoda. Although both results included Bivalvia, Malacostraca, and Gastropoda, there were still differences, mainly due to the advantages and limitations of two research methods. Microscopic examination technology identifies food species based on the parts of digested food, with simple operation and low requirements for the state of fecal samples, but some foods are easily completely digested, making it difficult to identify all food species (Pauling et al., 2016). However, some foods are easily completely digested, making it difficult to identify all food types. Based on the DNA present in feces for dietary analysis, it is not dependent on the undigested parts of food, and it is not limited by the type of food consumed, but it has relatively high requirements for the freshness of feces (Hawlitschek et al., 2018). Therefore, the comprehensive application of different research methods in wildlife dietary studies will yield more comprehensive findings.

Currently, the research on diet composition of relict gulls in different breeding grounds has focused on breeding period in this species, including the Ordos Plateau in Inner Mongolia (Liu et al., 2022) and the Hongjiannao wetland in Shanxi Province (Wang et al., 2022). The relict gull breeding in the Ordos Plateau mainly feeds on chironomid larvae and adults, damselfly larvae, toad tadpoles, and insects of the family Gryllidae (Liu et al., 2017), while relict gulls breeding in the Hongjiannao wetland mainly feeds on adult toads and tadpoles, chironomid larvae and adults, damselfly larvae, and scorpionflies (Wang et al., 2022). This indicates that there are differences on diet composition of relict gulls in different breeding grounds. Unlike previous studies on diet composition of relict gulls, our study focused on diet composition during the overwintering period. Based on preliminary comparisons of high-throughput sequencing results of 18S rRNA, it was found that the dominant classes in 2021 group and 2022 group were Polychaeta (2021 group: 2.41%; 2022 group: 71.72%) and Malacostraca (2021 group: 13.37%; 2022 group: 16.02%). Based on comparison between two groups, the unique dominant classes in 2021 group included Bivalvia, Gastropoda, unclassified_p_Dinoflagellata, Dinophyceae, and Trebouxiophyceae, while the unique dominant classes in 2022 group was Ostracoda. In summary, there are significant differences in diet composition of relict gulls during the breeding and overwintering periods, which reflects the characteristics of migratory birds that constantly adjust their diet structure based on external environments. In addition, there are significant differences in diet composition of the same habitat during the overwintering period, which are not only reflected in different food composition categories, but also in the proportion of the same type of food composition. In future research, a comprehensive study on nutritional ecology and dynamic changes in benthic resources of wild birds in the Tianjin coastal mudflat will help to further answer related questions.

Based on the analysis of high-throughput sequencing and microscopic examination technology, it was found that the abundance of Malacostraca was significantly positively correlated with the abundance of *Cetobacterium*, and the abundance of Gastropoda was significantly positively correlated with the abundance of *Catellicoccus*. These two findings were consistent across methods, indicating that different detection methods yielded consistent results. However, the difference lies in the fact that high-throughput sequencing technology showed that the abundance of Polychaeta was significantly positively correlated with the abundance of both *Romboutsia* and *Paeniclostridium*, while results based on microscopic examination technology showed that the abundance of Polychaeta was significantly negatively correlated with the abundance of both *Romboutsia* and *Paeniclostridium*. The reason for such difference may be that different methods have different detection depths of excavation for prey from fecal samples. In addition, the proportion of Polychaeta in fecal samples increased from less than 3% in 2021 group to 18.36% in 2022 group. Polychaeta, a class of animals that are the most abundant and relatively primitive in annelids, with more than 6,000 species, mostly living in the ocean (Kohlenbach et al., 2023), are also one of the main foods ingested by relict gulls during overwintering periods. Therefore, the inter-year comparison shows that the edible Polychaeta animals in coastal mudflats have increased.

Our research revealed disparities between microscopic examination technology and high-throughput sequencing technology in the analysis of relict gulls’ diet. These two methods exhibit their respective strengths and weaknesses when it comes to analyzing the composition of relict gulls’ diet based on fecal samples. Microscopic examination provides more direct data, while high-throughput sequencing captures a larger amount of prey DNA. Therefore, it is necessary to consider the results of both methods and combine them with the characteristics of relict gulls’ diet selection to obtain a more accurate understanding on diet composition. Furthermore, this study focuses specifically on the overwintering period’s diet composition for relict gulls. By comparing it with previous studies conducted during the breeding period, significant differences were observed in their diet composition between these two periods. This finding reflects migratory birds’ characteristic ability to continuously adjust their dietary structure according to external environmental factors. In future studies, a comprehensive study on nutritional ecology and dynamic changes in benthic resources of relict gulls in the Tianjin coastal mudflat will help to further answer related questions. Finally, this study also found the correlation between gut microbiota and diet composition. This lays the foundation for us to find markers of diet preferences in the future, and also provides a reference for obtaining the preference of migratory birds for prey through fecal contents.

## Conclusion

In summary, this study focuses on diet composition during overwintering periods as well as the potential relationship between diet composition and gut microbiota in wild relict gulls for the first time. Our results, combined with the results of other previous studies, can better present the characteristics of gut microbiota and diet composition from relict gulls throughout their life cycle, and explore the mechanisms of their adaptation to various environments, laying a foundation for better species conservation. However, the limitations of research methods should be considered. For example, the residual condition of debris in fecal sample may be affected by the degree of digestion. The high-throughput analysis primers selected in this study were 18 s rRNA region, and did not include plant endogenous ITS (internal transcribed spacer), resulting in some plant components not being fully detected. In the future, comprehensive comparison research on diet composition and gut microbiota of wild relict gulls living at multiple overwintering and breeding sites across years are required in order to provide integrative scientific reference for the ecological protection and management of this species.

## Data Availability

The datasets presented in this study can be found in online repositories. The names of the repository/repositories and accession number(s) can be found in the article/Supplementary material.
