# Supplementary material for: Inter-year consistencies and discrepancies on intestinal microbiota for overwintering relict gulls: correlations with food composition and implications for environmental adaptation
Source: Front Microbiol. 2024 Dec 9;15:1490413. doi: 10.3389/fmicb.2024.1490413 (PMC11683683; doi:10.3389/fmicb.2024.1490413)
Supplement: Supplementary file 1 [file Table_1.DOCX]

Supplementary Material

## Supplementary Tables：

**Supplementary Table 1. Details of samples used in experiment**

|  | Number of samples | Collection Time | Location |
| --- | --- | --- | --- |
| 2021 group | LR01-LR21 | 2021.11-2022.01 | 117.80273839, 39.13956585 |
| 2022 group | LR22-LR35 | 2022.12-2023.02 | 117.80273839, 39.13956585 |

**Supplementary Table 2. The mean relative abundance of the top 5 most abundant at phyla level.**

| **Sample group** | **Top five abundant phyla (%)** |
| --- | --- |
| 2021 group | Firmicutes(74.87) |
|  | Proteobacteria(10.90) |
|  | Fusobacteriota(5.14) |
|  | Actinobacteriota(3.26) |
|  | Desulfobacterota(1.34) |
| 2022 group | Firmicutes(75.92) |
|  | Proteobacteria(10.78) |
|  | Cyanobacteria(6.01) |
|  | Actinobacteriota(3.21) |
|  | Bacteroidota(1.11) |

**Supplementary Table 3. The mean relative abundance of the top 5 most abundant at genus level.**

| **Sample group** | **Top five abundant gerna (%)** |
| --- | --- |
| 2021 group | *Catellicoccus*(69.15) |
|  | *Cetobacterium*(5.13) |
|  | *unclassified_c_Gammaproteobacteria*(3.18) |
|  | *Breznakia*(1.90) |
|  | *Ilumatobacter*(1.09) |
| 2022 group | *Romboutsia*(20.29) |
|  | *Catellicoccus*(19.26) |
|  | *Breznakia*(17.01) |
|  | *unclassified_o_Chloroplast*(5.99) |
|  | *Psychrobacter*(4.49) |

**Supplementary Table 4. The alpha diversity index of fecal microbial composition**

| Samples | ace | chao | coverage | shannon | simpson |
| --- | --- | --- | --- | --- | --- |
| LR01 | 323.5765 | 289.1875 | 0.998105 | 1.453982 | 0.41624 |
| LR02 | 231.2215 | 167.3684 | 0.998642 | 0.457388 | 0.847188 |
| LR03 | 470.7193 | 290.6364 | 0.997737 | 0.205241 | 0.95072 |
| LR04 | 470.7059 | 139 | 0.998982 | 0.042889 | 0.990465 |
| LR05 | 538.674 | 450.5172 | 0.99686 | 1.437427 | 0.361498 |
| LR06 | 304.2113 | 279.15 | 0.997624 | 0.270444 | 0.937468 |
| LR07 | 338.1039 | 262.7143 | 0.997963 | 0.726131 | 0.743714 |
| LR08 | 688.7548 | 690.0667 | 0.996832 | 3.357709 | 0.136125 |
| LR09 | 530.5842 | 500.4 | 0.995927 | 0.639418 | 0.840818 |
| LR10 | 983.2495 | 945.2707 | 0.993296 | 3.355173 | 0.158871 |
| LR11 | 548.3649 | 390.5385 | 0.99686 | 1.09611 | 0.570261 |
| LR12 | 431.2238 | 293.3636 | 0.997539 | 0.357631 | 0.893273 |
| LR13 | 847.747 | 888.2289 | 0.993692 | 1.991012 | 0.386256 |
| LR14 | 729.8063 | 628.3 | 0.995418 | 1.950102 | 0.402378 |
| LR15 | 523.7859 | 372.5 | 0.99703 | 0.88475 | 0.70019 |
| LR16 | 775.4257 | 569.6545 | 0.995559 | 1.418018 | 0.437533 |
| LR17 | 1232.487 | 1000.755 | 0.992532 | 2.801397 | 0.307131 |
| LR18 | 1018.677 | 996.6522 | 0.992928 | 3.109972 | 0.201482 |
| LR19 | 1345.904 | 1315.124 | 0.992702 | 5.182396 | 0.012748 |
| LR20 | 483.2617 | 382.0189 | 0.996945 | 1.109357 | 0.65742 |
| LR21 | 699.4751 | 488.3684 | 0.995898 | 2.217574 | 0.187585 |
| LR22 | 608.8556 | 598.9634 | 0.996068 | 2.431382 | 0.207122 |
| LR23 | 1143.563 | 1124.143 | 0.992759 | 4.336857 | 0.038732 |
| LR24 | 612.348 | 599.6136 | 0.995814 | 2.525682 | 0.224579 |
| LR25 | 625.2295 | 611.8519 | 0.996012 | 2.895048 | 0.176469 |
| LR26 | 963.2958 | 954.024 | 0.993692 | 3.359967 | 0.12131 |
| LR27 | 710.9465 | 582.7143 | 0.995474 | 2.016045 | 0.307041 |
| LR28 | 658.5153 | 488.8421 | 0.996096 | 1.64438 | 0.416168 |
| LR29 | 917.0552 | 853.381 | 0.993551 | 2.898749 | 0.173688 |
| LR30 | 747.5433 | 738.6408 | 0.994484 | 2.634575 | 0.176826 |
| LR31 | 567.9653 | 583.5082 | 0.99604 | 2.324897 | 0.232894 |
| LR32 | 708.2819 | 695.2593 | 0.99522 | 2.67758 | 0.166576 |
| LR33 | 957.2129 | 941.5703 | 0.994145 | 3.816346 | 0.066083 |
| LR34 | 627.9053 | 465.6842 | 0.996492 | 1.911945 | 0.224408 |
| LR35 | 537.3104 | 532.0732 | 0.996606 | 2.538815 | 0.201519 |
