# Supplementary material for: Inter-year consistencies and discrepancies on intestinal microbiota for overwintering relict gulls: correlations with food composition and implications for environmental adaptation
Source: Front Microbiol. 2024 Dec 9;15:1490413. doi: 10.3389/fmicb.2024.1490413 (PMC11683683; doi:10.3389/fmicb.2024.1490413)
Supplement: Supplementary file 2 [file Data_Sheet_1.DOCX]

Supplementary Material

## Supplementary Figures

##
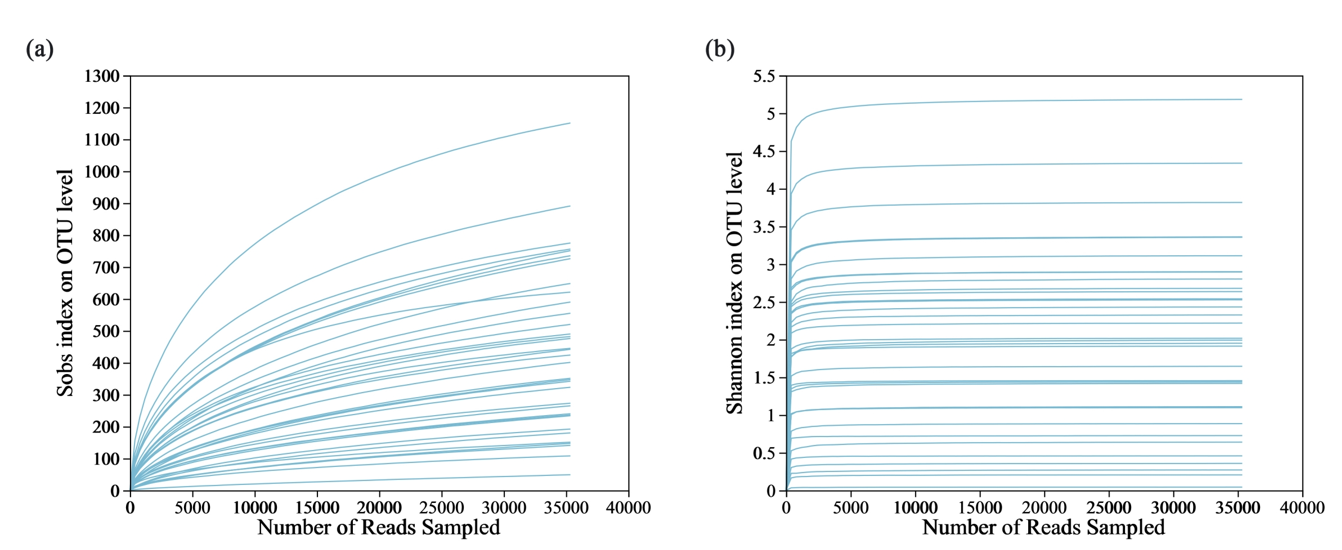


## Supplementary Figure 1. Rarefaction analysis of bacterial communities in gut of overwintering relict gulls based on Sobs index (a) and Shannon index (b)


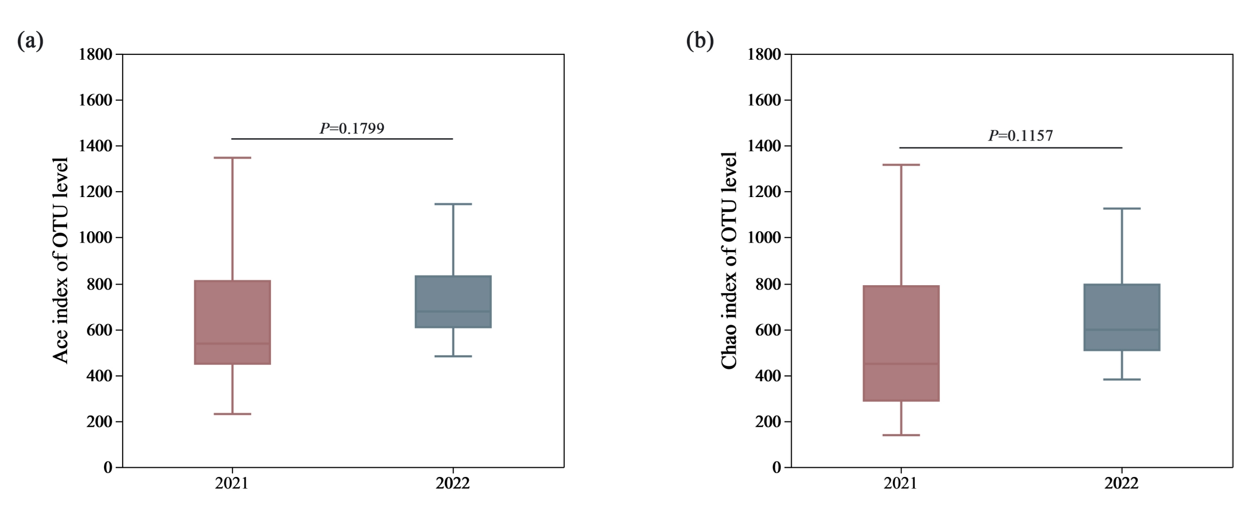


**Supplementary Figure 2.** The alpha diversity index of fecal microbial composition including Ace index (a) and Chao index (b)


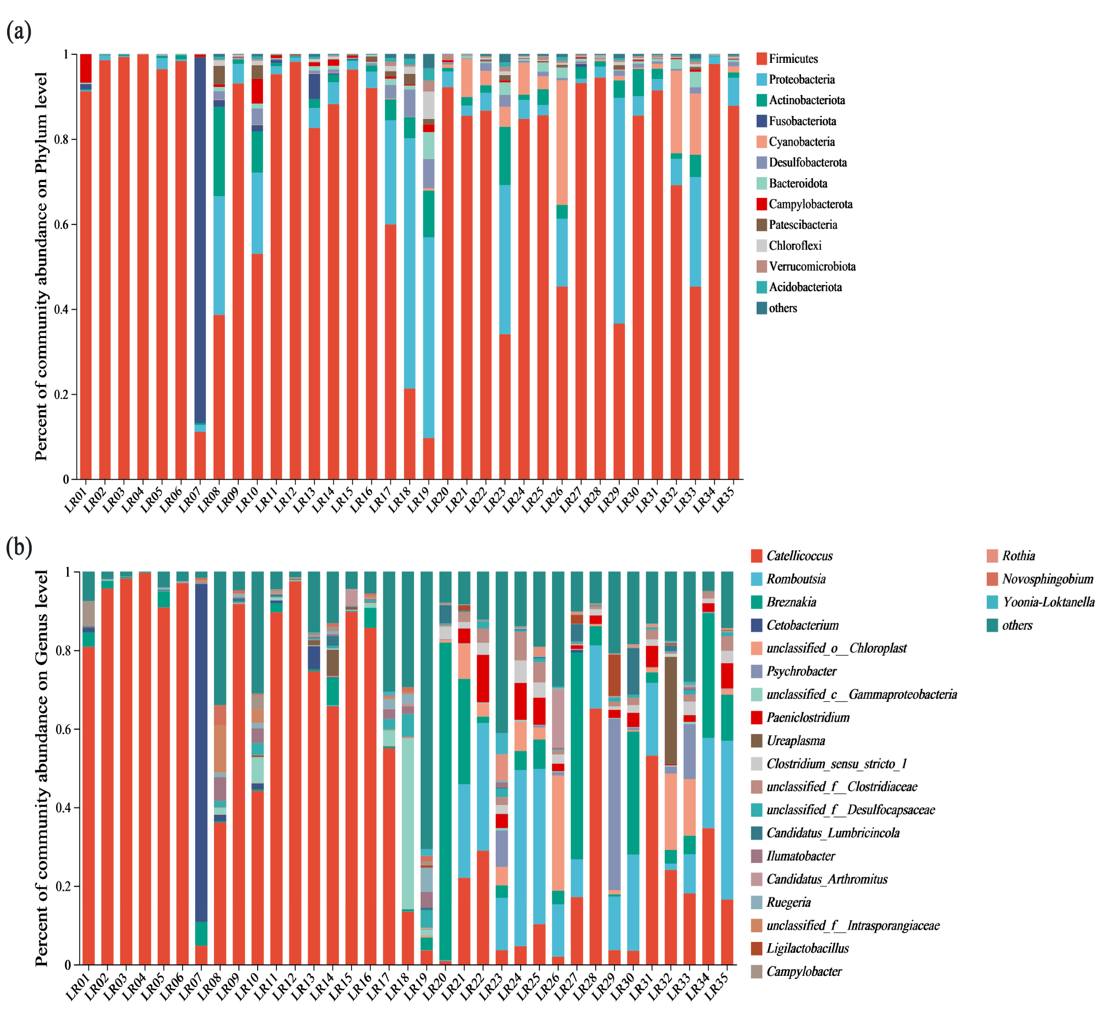


**Supplementary Figure 3.** Microbial composition of all fecal samples at the phylum (A), and genus(B) levels.

**
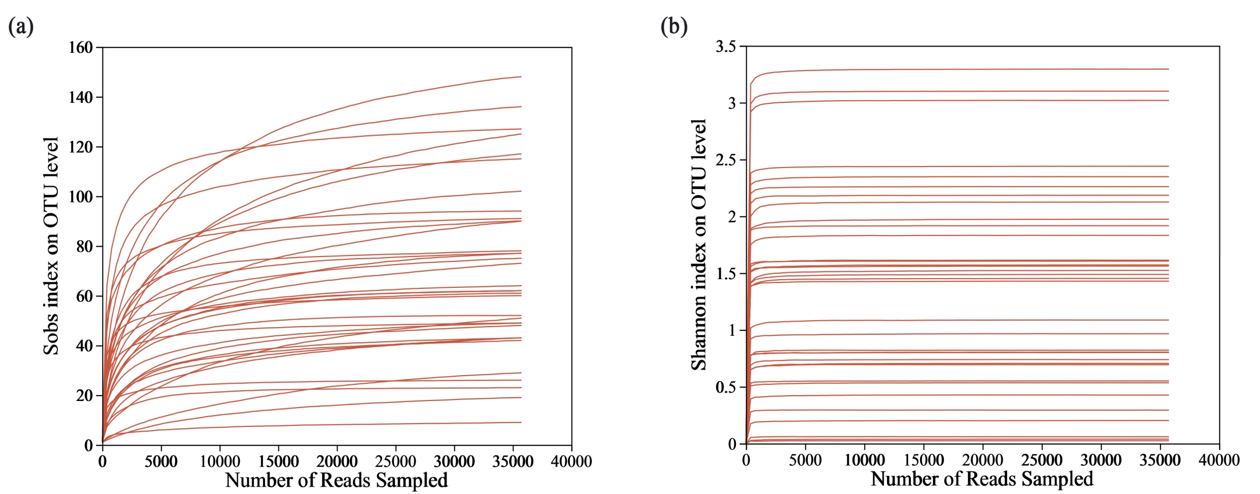
**

**Supplementary Figure4.** Rarefaction analysis of diet composition by high-throughput sequencing technology based on sobs index (a) and Shannon index (b)
